# Essential Hypertension: An Approach to Its Etiology and Neurogenic Pathophysiology

**DOI:** 10.1155/2013/547809

**Published:** 2013-12-09

**Authors:** Juan J. Bolívar

**Affiliations:** Departamento de Fisiología, Facultad de Medicina, Universidad Nacional Autónoma de México, Ciudad Universitaria, 04510 México, DF, Mexico

## Abstract

Essential hypertension, a rise in blood pressure of undetermined cause, includes 90% of all hypertensive cases and is a highly important public health challenge that remains, however, a major modifiable cause of morbidity and mortality. This review emphasizes that, from an evolutionary point of view, we are adapted to ingest and excrete <1 g of sodium (2.5 g of salt) per day and that essential hypertension develops when the kidneys become unable to excrete the amount of sodium ingested, unless blood pressure is increased. The renal-mean arterial pressure set-point model is briefly described to explain that a shift of the pressure natriuresis relationship toward abnormally high pressure levels is a pathophysiological characteristic of essential hypertension. Evidence indicating that this anomaly in the pressure natriuresis relationship arises from a sympathetic nervous system dysfunction is briefly formulated, and the most widely accepted pathophysiologic proposal to explain the development of this sympathetic dysfunction is described, with commentaries about novel action mechanisms of some drugs currently used in essential hypertension treatment.

## 1. Introduction

Hypertension, defined as a systolic blood pressure ≥140 mmHg and/or a diastolic pressure ≥90 mmHg, is one of the most common chronic diseases. The overall hypertension prevalence among the adult population was estimated at 26.4% in 2000 [1]; moreover it has been reported that this prevalence increased from 23.9%, in 1994, to 29.0%, in 2008, in the USA [2]; from 25.0%, in 1993, to 43.2%, in 2006, in Mexico [3]; and from 15.3%, in 1995, to 24.5%, in 2005, in Canada [4] among other countries. From this prevalence, it is evident that hypertension is a very important public health challenge because its complications, including cardiovascular, cerebrovascular, and renal diseases, are mayor causes of morbidity and mortality. Reducing blood pressure in individuals with hypertension prevents or attenuates these complications [5, 6].

Hypertension is due to specific causes in a small fraction of cases, but in the vast majority of individuals (*≈*90%), its etiology cannot be determined; therefore, the essential hypertension term is employed [5, 7]. Essential hypertension is currently understood as a multifactorial disease arising from the combined action of many genetic, environmental, and behavioral factors. Given the multifactorial nature of blood pressure homeostasis, any change in blood pressure as, for example, one due to a mutation, is likely to be compensated by feedback, complementary action, or change, in some other control mechanisms, in an effort to return blood pressure to normal. It is only when the balance between the factor(s) that tend to increase the blood pressure and those that try to normalize it is sufficiently disturbed, when the compensatory mechanisms fail to counteract the perturbation, that essential hypertension results [8]. A century of epidemiological, clinical, and physiological research in humans and animals has provided remarkable insights on the relationships existing between dietary salt (sodium chloride), renal sodium handling, and blood pressure. The evidence points to a causal link between a chronically high salt intake and the development of hypertension, when the kidneys are unable to excrete the ingested amount of sodium unless blood pressure is increased [9–11]. In conjunction with this primary causal factor, a number of adjunctive factors, such as obesity, diabetes, aging, emotional stress, sedentary life style, and low potassium intake, may increase the probability of developing hypertension [10, 12]. Hence, on a similar dietary salt, some individuals develop hypertension while others do not; and the probability to develop hypertension depends on the individual's weight of the hypertension's adjunctive factors.

## 2. Control of Blood Pressure by the Kidneys

**Figure 1:**
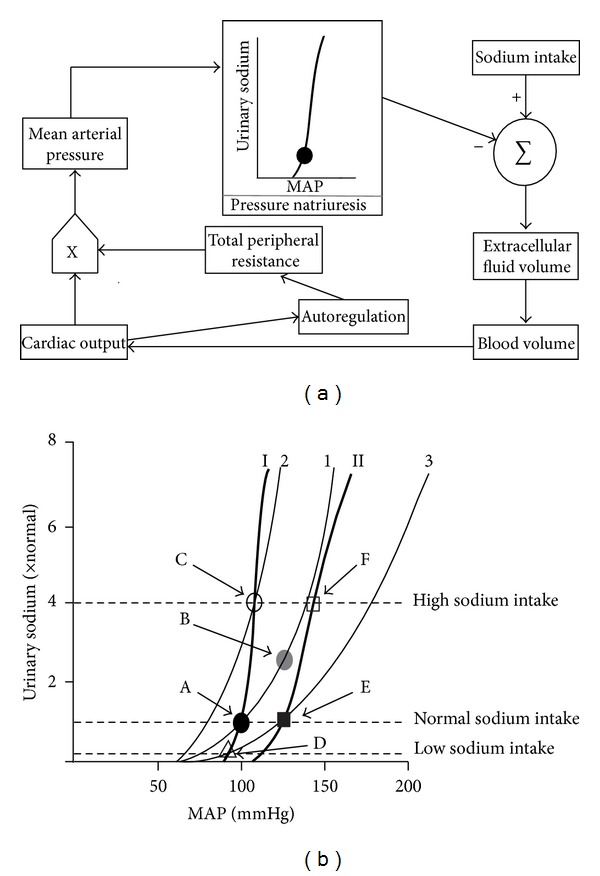
The renal-mean arterial pressure (MAP) set-point model as proposed by Guyton et al. [13–15]. (a) Basic renal-body fluid feedback mechanism for long-term regulation of blood pressure and body fluid volumes. (b) Normalized urinary sodium excretion is plotted as a function of the MAP to show the pressure natriuresis relationships, at different sodium intake levels, corresponding to the normal condition (acute renal function curves 1, 2, and 3 and chronic renal function curve (I)) and to a mild hypertension condition (chronic renal function curve (II) ). See further details in text.

The relative stability of arterial blood pressure leads to the conclusion that it is a highly controlled variable. Arterial pressure is maintained at the level satisfactory to ensure an adequate tissue perfusion. Baroreflexes and vasoactive hormones produce tight regulation over relatively short time spans [13]. Long-term regulation is, most generally, thought to be achieved through the renal fluid volume regulation mechanism. Regulation of mean arterial pressure (MAP) requires integrated actions of the physiological systems affecting its major determinants (Figure 1(a)). In the simplest formulation, determinants of MAP are approximated by Ohm's law modified for fluid dynamics (pressure = flow × resistance). Blood flow depends on cardiac output and blood volume, whereas resistance is primarily determined (as total peripheral resistance) by the contractile state of small arteries and arterioles throughout the body, which is itself determined by the tissues blood flow autoregulation mechanism. Blood volume depends on extracellular fluid volume (ECFV), which itself is determined by the total body sodium content. The latter depends on the balance (sodium equilibrium) between sodium intake and urinary sodium excretion (natriuresis; the main route of body sodium loss). Natriuresis is itself determined by the kidney's perfusion pressure, therefore the application of the pressure-natriuresis concept [8, 13–15]. For the purpose of the following discussion the term “normal sodium intake” refers to the current usual sodium intake (see Section 3, to realize to what extent this sodium intake is normal).

Long-term regulation of MAP is intimately associated with ECFV homeostasis. Sodium equilibrium is critical to ECFV, and the kidneys, as the principal route through which sodium is eliminated from the body, are therefore central to the long-term stability of MAP. This concept was expressed quantitatively in a systems analysis approach that predicts that the kidney acts as an overriding regulator of arterial pressure through a “renal-body fluid feedback” mechanism. A key component of this feedback is the pressure natriuresis or the effect of arterial pressure on renal sodium and water excretion, exemplified in acute and chronic renal function curves (Figure 1(b); thin and thick curves, resp.). Arterial pressure is set at the level required by the kidney to allow sodium and water excretion to match the intake (point A, Figure 1(b)). Basal-acute and normal-chronic renal function curves (curves 1 and I, resp.) coincide at this pressure level. Kidney perfusion studies show that, in the absence of a change in sodium intake, a rise in MAP (or renal perfusion pressure) is matched by increased renal sodium excretion (point B; sodium excretion exceeds intake), or pressure natriuresis, which reduces ECFV and cardiac output and returns MAP to normal. Therefore, disturbances that tend to increase arterial pressure, such as increased peripheral vascular resistance, would cause only a transient increase in arterial pressure, because they would also provoke increased renal sodium excretion. Conversely, if MAP falls below the control level, a reduced renal sodium excretion (antinatriuresis) increases ECFV and MAP. Hence, the kidney strives to protect against perturbation from the sodium equilibrium set point, and sodium balance and MAP are maintained by a feedback system displaying infinite gain. That infinite gain is invoked to explain the fact that when sodium intake is increased, renal sodium excretion is similarly increased (point C), in response to a very small increase (if any) in MAP, to attain sodium equilibrium. Again, acute and chronic renal function curves (curves 2 and I, resp.) coincide at the new pressure level, because acute renal function curve 2 corresponds to the kidney function influenced by all the regulatory mechanisms triggered by the increased sodium intake (regulatory mechanisms acting to decrease renal tubular sodium reabsorption); and also because the normal-chronic renal function curve I corresponds to the pressure natriuresis relationship observed after all regulatory mechanisms acting at any sodium intake level have influenced the kidney function. When sodium intake is decreased, renal sodium excretion is similarly decreased (point D), in response to a very small decrease in MAP. As expected, acute renal function curve 3 (regulatory mechanisms acting to increase renal tubular sodium reabsorption) and normal-chronic renal function curve I coincide at the slightly decreased MAP level. If this feedback mechanism is valid, hypertension results from a shift in the renal-pressure natriuresis function to the right (chronic renal function curve II), so that a higher pressure is required to attain sodium balance on a normal sodium intake (point E). In this condition, the acute renal function curve 3 and the abnormal-chronic renal function curve II coincide at a hypertensive MAP level. Hence, the acute renal function curve 3 (which corresponds to a situation with regulatory mechanisms acting to increase renal tubular sodium reabsorption) is operative, even when sodium intake is normal. In the presence of hypertension, if sodium intake is increased, a higher than normal MAP increase is necessary to obtain sodium balance (point F) [8, 13–15]. With the intrinsic kidney function being normal, in the early stage of essential hypertension, an abnormal pressure natriuresis relationship can only result from an abnormal regulation of the kidney function.

## 3. Salt Intake

If the start of human evolution is arbitrarily set at the beginning of the Paleolithic, during 3 million years, the ancestors of humans, like all other mammals, ate a diet containing little sodium and much potassium: some 0.6 g and 7 g per day, respectively, a Na^+^/K^+^ relationship close to 0.09 [22]. In the Stone Age, the average life span was approximately 30 years. During these times, traits that worked to increase blood pressure with increasing stress would be favorable for survival: people who could easily elevate their blood pressure to provide sufficient blood to skeletal muscles and major organs would have a survival advantage when attacked by enemies or wild animals. Thus, the ability to easily increase blood pressure is a characteristic that might have conferred an evolutionary advantage until modern times [10]. Blood pressure is directly proportional to total body sodium content. As sodium intake is limited in natural foods, physiological mechanisms to promote sodium ingestion and to prevent sodium loss into urine would have been established early in human evolution [23]. To promote sodium ingestion, sodium appetite is a motivated behavioral state, arising in response to sodium deficiency that drives humans to seek and ingest food and fluids containing sodium [24]. To prevent sodium loss, the most powerful mechanism is the renin-angiotensin-aldosterone system (RAAS), which controls kidney's tubular sodium reabsorption and which is maximally activated in people with a minimal sodium intake [10]. In addition, sodium depletion or emotional stress activates the sympathetic nervous system, which, acts mainly via stimulation of the RAAS and further prevents urinary sodium loss [25, 26]. Besides sodium appetite, evolution has provided humans with a pleasant liking of salt taste, which motivates man to ingest sodium in excesse of need, when it is available [24].

About 8000 years ago, humans discovered that salt could be used to preserve food and developed sophisticated techniques for salt production [27, 28]. Humans then satisfied their innate taste for salt and have been adding it to food ever since [27, 29]. Salt then became of great economic importance as it made it possible to preserve food, allowing the development of cities. Salt was the most taxed and traded commodity in the world [30, 31]. Romans in the first century AD used salt as money “salarium” and considered it synonymous of health “salus”, “salubritas” and more necessary than gold [27, 29]. Hence, due to its use in food preservation, salt consumption was high in historical times, some 10–50 g/day per person, in Europe in the 18th century, mainly from cured meat and salted fish and meat (implying that salt might be partially discarded with the cooking water) [27]. However, with the invention of the deep freezer and the refrigerator (at the end of the 19th century), salt was no longer required as a preservative. Salt intake had been declining, but with the recent large increase in consumption of highly salted (and potassium poor) processed food, salt intake is now increasing towards levels similar to those of the 18th century and is approximately 9–12 g/day (3.6–4.8 g of sodium, that is, 6 to 8 times more than our evolutionary sodium intake), with a sodium/potassium intake ratio >2 (more than 20 times our evolutionary ratio), in most countries in the world [22, 31, 32].

**Figure 2:**
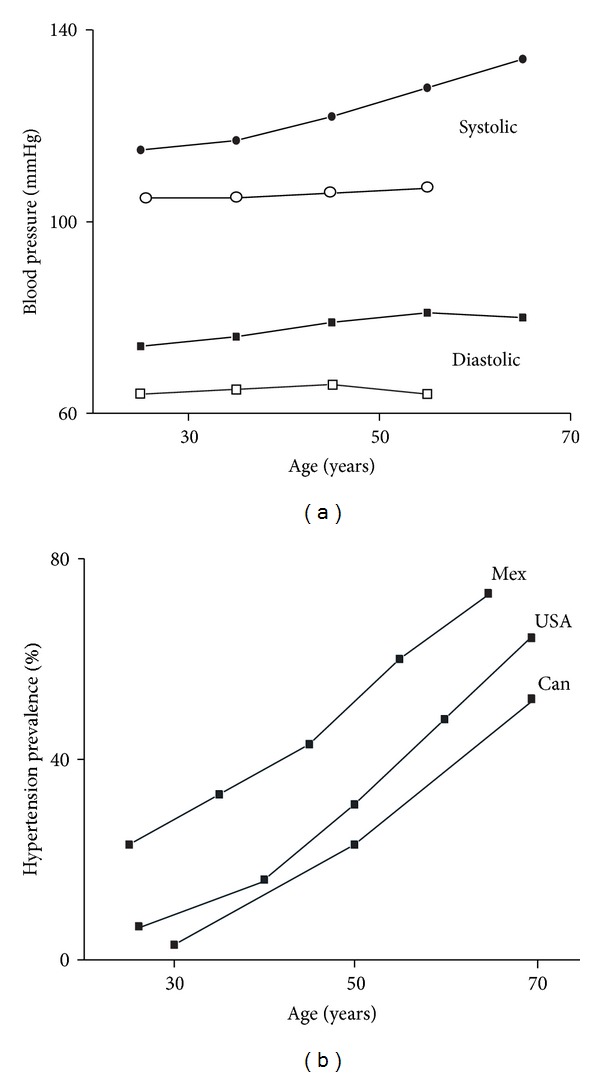
Blood pressure and hypertension during the adulthood. (a) Systolic and diastolic blood pressure mean values (represented in 10-year age group intervals) as a function of age in nonacculturated populations (which do not add salt to food, open symbols) and in an acculturated (the Mexican) population (which does add salt to food, closed symbols). Data adapted from [3, 16]. (b) Hypertension prevalence as a function of age in the populations from Mexico (Mex), the United States of America (USA, both represented in 10-year age group intervals, with the exception of the first point of the USA graph, which represents a 17-year group interval), and Canada (Can, represented in 20-year age group intervals). Data adapted from [3, 17, 18].

Although preference for salty-tasting food and prevention of sodium loss may once have conferred an evolutionary advantage, ingestion of excessive amounts of sodium now results in chronic hypertension [10, 11, 24]. Many large observational epidemiological investigations conducted worldwide link high salt intake and hypertension [30, 33]. In one of the first global studies on sodium intake [32, 34], 24 h urine sodium and urinary sodium/potassium relationship were positively associated with blood pressure as well as the increase in blood pressure with age. Furthermore, populations with low average daily sodium intake (some tribal societies which do not add salt to the food) had relatively low blood pressure and very little or no increase in blood pressure with age [16]. Hypertension was fairly uncommon in these societies, but individual's blood pressure rose after migration to an urban environment. However, migration involves more change than just a change in salt intake, because other factors, such as mental stress and changes in physical activity and diet, may contribute to the rise in blood pressure [33].  Figure 2(a) shows that in acculturated populations (as the Mexican population), which add salt to the food, systolic and diastolic blood pressure increase with age and that this increase does not occur in nonacculturated populations. In the same way, in acculturated populations (as those of Canada, Mexico, and USA), hypertension prevalence increases with age (Figure 2(b)) [3, 17, 18]. However, blood pressure increase with age is higher in urban than in rural environments, reflecting the environmental influence on blood pressure [35]. On the other hand, in two clinical studies performed on some 200 individuals in which, within their usual diet, dietary sodium intake was randomly and sequentially adjusted at low (1.15 g/day), intermediate (2.30 g/day), and high (3.45 g/day) levels, during three 30-day periods, a positive relationship was found between blood pressure and sodium ingestion, supporting the conclusions of the population studies described above [36, 37].

In contrast to sodium, potassium was abundant in the fresh food that made up the stone age diet; but, in modern times, diets have shifted drastically to processed foods, reducing potassium intake [22, 31]. A low potassium diet induces sodium retention and increases blood pressure [38]. On the contrary, potassium supplementation promotes natriuresis and decreases blood pressure [39].

Epidemiological studies worldwide suggest that the optimal daily intake of salt is 5-6 g [40] and some 3.5 g of potassium [41], roughly half and twice of the current average intake of sodium and potassium, respectively. However, because humans have evolved with sodium deficiency for a long time, we have developed a powerful hedonistic taste for salt. This innate desire for salty foods, to which cultural and social habits have superimposed, makes it very hard to drastically reduce sodium intake [10, 42]. Nonetheless, we must realize that the human body is not equipped to handle the unnatural amount of sodium present in our current diet; hence, hypertension, to some extent, may be classified as a disease of “affluence” [11, 42].

## 4. Abnormal Regulation of the Kidney Function

At the end of the 19th century, the renal sympathetic nerves were known to contain fibers which upon stimulation decreased renal blood flow and urinary flow rate. It was also known that renal blood flow and urinary flow rate increased after renal sympathetic nerves transection [43]. In the early decades of the 20th century, faced with the high mortality of severe hypertension and the absence of effective pharmacological therapy, a number of operations on the sympathetic nervous system, such as radical splanchnicectomy, were devised in an attempt to lower blood pressure. By the late 1960s, most of the available antihypertensives, which by then had been developed, antagonized the sympathetic nervous system. The potency and clinical usefulness of these drugs helped to sustain the argument that the sympathetic nervous system was important in the pathogenesis of essential hypertension [44].

The sympathetic nervous system exerts a basal excitatory activity over the kidney. Increases in this activity result in (1) increase in renin secretion, (2) increase in renal tubular sodium reabsorption, and (3) renal vasoconstriction. Experimental studies established the concept that subvasoconstrictor levels of renal sympathetic nerve activity can produce increased renin secretion and increased tubular sodium reabsorption (without changes in renal blood flow and glomerular filtration rate), which result in a shift in the renal-pressure natriuresis function to the right, so that a higher than normal arterial pressure is required to attain sodium balance [12]. There is now convincing evidence that sympathetic nervous system activity is increased in patients with essential hypertension [12, 43]. Within this evidence is the finding that normotensive young men with a family history of hypertension have a higher sympathetic nerves activity than those without a family history [45]. During mental stress, sympathetic nerves activity and blood pressure increase in normotensive offspring of parents with essential hypertension, but do not increase in those with normotensive parents [46, 47]. One-third of patients with borderline hypertension display so-called hyperkinetic circulation, characterized by an elevation in resting heart rate combined with a high cardiac output and an increase in the circulating plasma level of the adrenergic neurotransmitter norepinephrine [44]. In the same way, renal sympathetic nerve activity is augmented two- to threefold (on average) in young patients (<45 years) with essential hypertension [48, 49]. These patients also show an increased renin release and plasma renin activity. On the other hand, in patients with resistant hypertension, responding inadequately to concurrent treatment with multiple antihypertensive drug classes, radiofrequency ablation of the renal sympathetic nerves lowers blood pressure remarkably [44]. Nowadays, it is deemed that a neurogenic origin (sympathetic activation) of essential hypertension could account for up to 50% of all cases of high blood pressure [12]. However, increased sympathetic nerves activity is most clearly expressed in the early stages of hypertension development and is less consistent as the time passes [48, 50]. Once chronic hypertension is installed, and after the early stages of essential hypertension, hypertensive blood pressure levels may be maintained, even in the absence of an increased renal sympathetic nerve activity, mainly by a secondary kidney disease, characterized by glomerulosclerosis, interstitial fibrosis and proteinuria [51, 52].

## 5. Origins of Sympathetic Nervous System Activation in Essential Hypertension

The specific causes of the increased sympathetic activity in essential hypertension are only partially known. Genetic influences (a family history) are evident, and behavioral (as salty food preference), psychosocial (as mental stress) and lifestyle (as physical inactivity) factors appear to be involved [12, 50]. Of prime importance, no doubt, is obesity. The prevalence of hypertension in middle-age obese subjects is 40–50%. Obesity increases the sympathetic (including the renal sympathetic) nervous system activity through the high sodium intake-related mechanisms that will be discussed below and through other mechanisms, such as hyperleptinemia, that will not be reviewed in this paper [53, 54]. Likewise, although the prevalence of hypertension increases with aging and 60% of all adults aged 60–69 years are hypertensive, owing to the pathogenic factors associated with an increased sympathetic nervous system activity in the elderly, such as high dietary sodium intake and increasing obesity, and only the former will be discussed here [55, 56]. On the other hand, clinical and epidemiological studies indicate the importance of chronic mental stress in the pathogenesis of essential hypertension [25, 57]. Hypertensive subjects may decrease their blood pressure with a meditation program [58, 59]. Psychosocial stress can increase the activity of the sympathetic nervous system by potentiating the neural mechanisms activated by a high salt intake [60]. Race and ethnicity may also influence the predisposition to the sensitivity of blood pressure to salt. Black Africans have a higher prevalence of hypertension and more frequent severe hypertension; they also have a greater blood pressure sensitivity to salt intake than do people of other ethnic origins [25, 61]. Physical inactivity also appears to be important [12]. Aerobic fitness and physical activity are each inversely related to the development of hypertension [62]. Aerobic exercise training in sedentary normotensive and hypertensive people reduces blood pressure and renal and muscle sympathetic nerves activity [63, 64].

**Figure 3:**
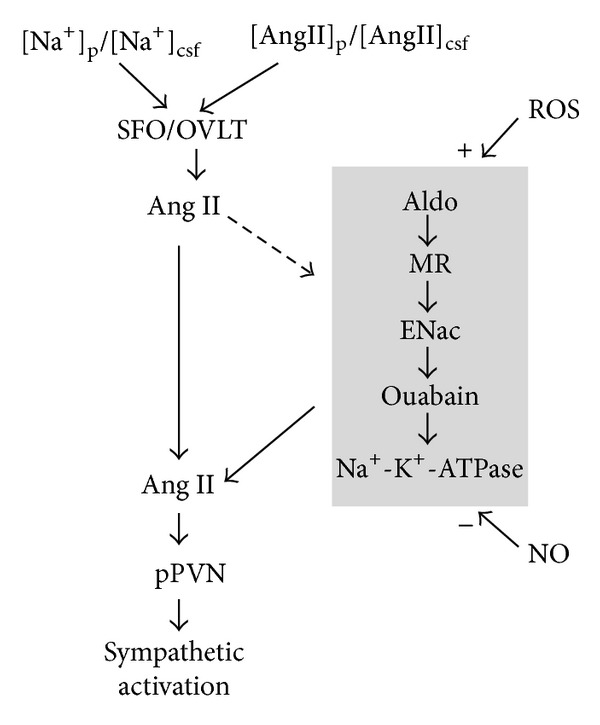
Diagram of the central nervous system pathways that may be activated by an increase in one or more of the following agents: circulating [Na^+^]_p_, [Na^+^]_csf_, circulating [Ang  II]_p_ and [Ang  II]_csf_. Left: agents, acting primarily on the sensorial SFO and OVLT, acutely activate a sympathoexcitatory angiotensinergic pathway that increases the activity of the presympathetic pPVN neurons. Right: chronic increase in these agents also activates, via the SFO and the OVLT, a neuromodulatory pathway, which then maintains enhanced excitatory angiotensinergic activity on the presympathetic pPVN neurons, leading to sustained sympathetic activation and hypertension. See further details in text. Diagram adapted from [10, 19–21].

In hypertensive individuals with their usual salt intake (9–18 g/day) the concentrations of plasma sodium ([Na^+^]_p_) and cerebrospinal fluid (CSF) sodium ([Na^+^]_csf_) are slightly increased (by 0.5–3 mM), as compared with values observed in the same individual on a low (3-4 g/day) salt intake. The same variation on [Na^+^]_p_ (and probably in ([Na^+^]_csf_) in normotensive individuals is observed in the same circumstance [65, 66]. Similar changes in [Na^+^]_p_ and in [Na^+^]_csf_ are observed in animal models of hypertension, on a high sodium diet [67, 68]. Studies performed in animal models allow proposing that these increased [Na^+^]_p_ and/or [Na^+^]_csf_ activate brain's sodium/osmoreceptors, located mainly at the hypothalamic lamina terminalis, to trigger sympathoexcitation [67, 69]. These osmoreceptors do not appear to reset significantly with prolonged change in osmolality and therefore can provide a sustained signal to chronically increase sympathetic tone [69]. Similarly, water deprivation-induced increases in osmolality act, at least in part, in the brain to promote sympathoexcitation and support blood pressure [70, 71]. The lamina terminalis comprises three structures aligned in the anteroventral region of the third ventricle. Two of them, the subfornical organ (SFO) and the organum vasculosum of the lamina terminalis (OVLT) are located outside the blood-brain barrier and are sensitive to humoral factors, such as Na^+^ and angiotensin II (Ang II). The third structure, the median preoptic nucleus (MnPO), located within the blood barrier, has reciprocal connections with the two other structures and integrates the humoral sensory information raised in the SFO and OVLT [72–74]. SFO and OVLT increase their activity in response to an increase in [Na^+^]_p_ and/or [Na^+^]_csf_, as well as to an increase in plasma and/or CSF Ang II concentration ([Ang  II]_p_ and [Ang  II]_csf_, resp.) [50, 67, 71, 72, 75]. Diverse studies have shown that SFO and OVLT express Na^+^ conducting nonselective cationic channels (which serve as extracellular Na^+^-levels sensors) and the Ang II type 1 receptor (AT_1_ receptor), whose expression is enhanced by a high sodium diet and by blood-borne Ang II [73, 76–82]. The latter may explain, at least partially, the antihypertensive and sympathoinhibitory actions of systemic AT_1_ receptor blockers, such as losartan or valsartan [83–86]. From the SFO and OVLT, perhaps after integration in the MnPO, signals are conveyed from the lamina terminalis to the presympathetic hypothalamic parvocellular neurons of the paraventricular nucleus (pPVN), mainly through Ang II-AT_1_ receptor mediated synapses, though some participation of glutamatergic synapses at the pPVN level has been described (Figure 3, left) [73, 87–90]. The angiotensinergic nature of most synapses involved in this conduction may explain, at least partially, the antihypertensive and sympathoinhibitory actions of systemic angiotensin-converting enzyme type 1 (ACE_1_) inhibitors, such as enalapril or captopril [70, 85, 91–93]. At this point, it is necessary to mention that all known components of the RAAS, including the precursor and enzymes required for the production and metabolism of angiotensin peptides and specific AT_1_ and AT_2_ receptors, as well as aldosterone (Aldo, which may even cross the blood-brain barrier) and mineralocorticoid receptor (MR), have been identified in the brain [94–97]. It is thought that the direct SFO-, OVLT-pPVN pathway described above participates mainly in rapid sympathetic responses to changes in [Na^+^]_p_ or [Na^+^]_csf_ or [Ang  II]_p_ or [Ang  II]_csf_ and in cardiovascular reflexes or acute psychogenic stress response [73, 74, 98, 99]; however, in generating the chronic sympathoexcitation observed in essential hypertension, another indirect or neuromodulatory SFO-, OVLT-pPVN pathway is involved (Figure 3, right) [10, 19–21, 94]. This neuromodulatory pathway is characteristically dependent on protein phosphorylation and changes in protein expression, and its activation promotes increases in renin, ACE_1_, AT_1_ receptors, Aldo synthase, and NADPH oxidase and decrease in neuronal nitric oxide (NO) synthase in the hypothalamus [100–104]. This polysynaptic pathway is slowly activated (days or weeks) by a chronic increase in [Na^+^]_p_ and/or [Na^+^]_csf_ or in [Ang  II]_p_ and/or [Ang  II]_csf_ [10, 19–21, 105] and may be inhibited by the systemic ACE_1_ inhibitors and AT_1_ receptor blockers, mentioned above, as well as by systemic spironolactone, an Aldo antagonist [77, 85, 93, 106, 107]. This neuromodulatory pathway appears to rise from SFO and OVLT projections directed to the magnocellular neurons of the paraventricular and supraoptic nucleus of the hypothalamus, but its exact anatomical location is uncertain [21, 77, 94, 108]. Angiotensinergic conduction, involving Ang II and AT_1_ receptors, is present at least at the beginning (SFO and OVLT) and end (pPVN) of this pathway [108–111], but it involves the sequential participation of diverse neuromodulatory agents, receptors, and ion transport mechanisms, such as Aldo, MR, benzamil-blockable epithelial Na^+^ channels (ENaC), endogenous ouabain-like compounds (ouabain), and ouabain-sensitive Na^+^-K^+^-ATPase [10, 19–21, 101, 104, 105]. The activity of this pathway is regulated by the balance between the inhibitory influence of NO and the stimulatory influence of reactive oxygen species (ROS), such as superoxide and peroxynitrite; however, as a consequence of the activation of this pathway by the factors mentioned above, production of Ang II and Aldo increases, and this increase, by promoting ROS generation and inhibiting NO synthesis, shifts the NO/ROS balance to an enhanced excitation [10, 101, 105, 112–114]. This abnormal balance may be corrected, at least to some extent, by chronic systemic administration of the gradually, and long-acting dihydropyridine calcium channel blockers azelnidipine, cilnidipine, and amlodipine; and the antidyslipidemic agents simvastatin and pravastatin (chronic peripheral administration of both kind of drugs appears to result in gradual access of drug to the central nervous system) and by regular exercise training; each of them increases NO and decreases ROS, in the brain, thereby partially explaining the antisympathetic and antihypertensive actions of these therapeutics and life style agents [115–124]. The activity of the neuromodulatory pathway maintains an enhanced activity of the pPVN neurons, which increase the sympathetic nerve activity through a direct pathway, mainly vasopressinergic, to the sympathetic preganglionic neurons located at the intermediolateral (IML) cell column of the spinal cord, and through an indirect pathway (vasopressinergic, angiotensinergic, and glutamatergic) to the presympathetic neurons in the rostral ventrolateral medulla (RVLM) [50, 74, 125–128]. In turn, the presympathetic neurons in the RVLM activate the IML sympathetic preganglionic cells through a glutamatergic pathway [129–131]. Hence, activation of the neuromodulatory pathway maintains an increased activity of the pPVN, the RVLM, and the IML presympathetic and sympathetic neurons, leading to sympathoexcitation and hypertension.

## 6. Conclusion

Control of blood pressure requires complex integration of regulatory mechanisms across multiple physiological systems. A sustained increase in arterial pressure therefore reflects a failure of one or more of these controls. The hallmark of essential hypertension, and of all types of chronic hypertension from whatever origin, is an abnormal renal-pressure natriuresis relationship, which is shifted to the right, so that sodium equilibrium is obtained at a higher than normal pressure level. This review has emphasized the causal link between an abnormal high salt intake and essential hypertension. A high salt intake (9–12 g/day) is a pleasant component of our current normal diet, but it is abnormal from an evolutionary point of view. This high salt intake induces a slight increase in [Na^+^]_p_ and/or [Na^+^]_csf_, which, when sensed by the lamina terminalis sodium/osmoreceptors, triggers, in susceptible individuals, a hypothalamic neuromodulatory signaling chain, activating the sympathetic nervous system. An increased sympathetic nervous system excitatory activity toward the kidney results in increased renin secretion and renal tubular sodium reabsorption and, consequently, in a shift to the right of the pressure natriuresis relationship. Hence, in essential hypertension, the abnormal pressure natriuresis relationship is due to an increased activity of the renal sympathetic nerves. However, this increased renal sympathetic activity is most clearly expressed in the early stages of essential hypertension development and is less consistent as time passes. This review has focused on two of the main etiological and pathophysiological mechanisms responsible for the onset and maintenance of uncomplicated essential hypertension. Once chronic hypertension is installed, it may be maintained, even in the absence of an increased renal sympathetic nerves activity, mainly by a secondary kidney disease.
